# Complementary approaches to measure predation pressure on butterfly populations in Australia

**DOI:** 10.1098/rsos.251507

**Published:** 2025-10-29

**Authors:** Hansani Sathsara Sandukalani Daluwatta Galappaththige, Marilia Fernandes Erickson, Donald James McLean, Kiara Laëtitia L'Herpiniere, Georgina Erika Binns, Liisa Hämäläinen, Louis G. O'Neill, James Kevin Douch, David W. Kikuchi, Hannah M. Rowland, Johanna Mappes, Marie E. Herberstein

**Affiliations:** ^1^ School of Natural Sciences, Macquarie University, Sydney, New South Wales, Australia; ^2^ Department of Biological and Environmental Science, University of Jyväskylä, Jyväskylä, Finland; ^3^ Australian Wildlife Conservancy, Subiaco East, Australia; ^4^ Department of Integrative Biology, Oregon State University, Corvallis, OR, USA; ^5^ Department of Evolution, Ecology and Behaviour, Institute of Infection Veterinary and Ecological Sciences, University of Liverpool, Liverpool L69 7ZB, UK; ^6^ Organismal and Evolutionary Biology Research Programme, University of Helsinki, Faculty of Biological and Environmental Sciences, Helsinki, Uusimaa, Finland; ^7^ Museum of Natural History Hamburg, Leibniz Institute for the Analysis of Biodiversity change, Martin Luther King Square 3, Hamburg, Germany; ^8^ University of Hamburg Department of Biology, Hamburg, Germany

**Keywords:** predator–prey interactions, bird-attacked butterfly wing, butterfly replica, avian predators, bird surveys

## Abstract

Predation pressure is a major force driving the evolution of anti-predatory traits, yet quantifying its intensity in the wild remains difficult. In this study, we combined three complementary methods to evaluate predation risk in Australian butterfly communities: assessing wing damage on wild butterflies, monitoring attacks on artificial butterfly replicas, and surveying bird communities. Across eight sites in Sydney, Australia, we recorded wing damage on 1070 free-living butterflies from five families, assessed attacks on 1600 butterfly replicas, and surveyed local bird diversity. Of the wild butterflies, 807 showed wing damage, with 169 individuals (16% of all butterflies) exhibiting patterns consistent with bird attacks. Among replicas, 114 (7%) showed evidence of predation, of which 31 (2% of all replicas) were likely to be attacked by birds. Predation on wild butterflies was most strongly associated with bird community composition, bird density, and butterfly size (wingspan), while replica attacks were influenced primarily by bird community composition and density. Our results suggest that butterfly wing damage and replica attacks data provide complementary, but not interchangeable, insights into predation risk. When integrated, they offer a more robust picture of true predation pressure. Our results underscore the critical role of bird community structure in shaping predation risk—an important consideration for any method used to assess predation in natural prey communities.

## 1. Introduction

Predation is a major selective force driving the evolution of anti-predatory strategies, influencing not only individual behaviour and morphology, but also population distribution patterns, and community structure [1–5]. Yet accurately measuring predation pressure in the wild remains challenging, as direct predation events are rare, brief and often difficult to observe systematically [6].

Several field and experimental methods have been developed to address these challenges, including direct observation of predation events, the analysis of predator-inflicted damage on prey (either natural or artificial), and the identification of prey remains using molecular or morphological tools (see [7], for a review). These approaches are often complemented by predator surveys, which help contextualize attack rates in relation to predator densities and community composition. Laboratory-based feeding trials, where predators’ preferences among prey types are tested, can also serve as proxies for relative predation pressures in natural contexts [7].

Butterflies are well-suited as model prey in predation studies due to their conspicuous colouration and diverse anti-predator traits, including crypsis, disruptive patterns, eyespots, and conspicuous warning signals [8–12]. These traits are widely accepted to have evolved in response to visually orientated predators, especially birds [13–17]. As a result, the butterfly-prey–bird-predator system offers a robust framework for investigating the ecological and evolutionary consequences of predation pressure.

While direct observations of bird attacks on butterflies provide valuable insights, they are inherently limited in scope—typically focusing on a small number of predator or prey species, and sometimes unusual contexts (e.g. aggregations, puddling sites [18–20]). For example, Calvert *et al*. [18] found that 37% of observed attacks by birds on Monarch butterflies (*Danaus plexippus*) led to rejection after damage. Burger & Gochfeld [19] observed Smooth-billed Ani (*Crotophaga ani*) preying on Sulphur butterflies (*Phoebis trite* and *Aphrissa statira*) in Brazil, with approximately 8% of butterflies escaping after being captured. While Chai & Srygley [21] and Pinheiro & Cintra [20] recorded feeding behaviours in tropical bird species. Most recently, Stefanescu [17] identified several bird species feeding on Painted Lady butterflies (*Vanessa cardui*) across the Palaearctic–Afrotropical region. Despite their value, such observations are logistically difficult and represent only a fraction of butterfly–bird interactions. Consequently, ecologists have increasingly turned to indirect approaches such as assessing wing damage on live butterflies, measuring attack rates on artificial replicas, and conducting bird community surveys [13,22,23].

Each method provides unique advantages and limitations. Wing damage analysis provides a useful proxy for unsuccessful predation [18,19,24], especially when beak-shaped marks are visible [25]. Wing attack frequency, location, and symmetry can offer insight into attack context, e.g. flight versus rest (single wing damage versus symmetric damage to both wings [23,26]). Collecting damaged butterflies also yields additional data on butterfly diversity, abundance, and sex ratio. However, this method cannot detect successful predation and may introduce a size bias if birds attack larger butterflies but eat smaller ones entirely. It also cannot be used to identify the species of bird predator [27]. There will also probably be some sampling bias with this method, as butterfly catching may not be a perfect representation of the butterfly community, since species differ in their activity and hence in how difficult they are to detect and capture (see electronic supplementary material, table S1 for more pros and cons).

Artificial butterfly replicas, typically made of clay, are widely used in ecological studies to estimate relative predation risk across different phenotypes [22,28]. These models offer a standardized, replicable, and cost-effective approach for testing hypotheses about the visual components of prey appearance, such as colouration or patterning. However, they lack movement, scent and other cues, and their stationary, open-wing posture may not reflect real resting behaviour [29,30]. Still, they offer valuable and scalable insights, especially when used in combination with other methods.

Bird surveys are often considered weak proxies for predation pressure relative to direct observations [27], yet they provide essential context for interpreting butterfly predation patterns. As the primary visual predators of butterflies [17,20,21,31], birds’ abundance and diversity influence predation risk. Bird survey data can be used to validate or explain observed attack rates on butterflies and replicas (see electronic supplementary material, table S1 for more pros and cons).

Each method captures a different aspect of the predator–prey interaction: wing damage reflects real but failed attacks; replicas reveal potential visual risk; bird surveys assess predator communities. Used in isolation, each has limitations, but combined, they offer a more complete picture of predation pressure and the spatial and temporal variation in predation risk, which can be scaled to encompass multiple prey types and habitats [32].

In this study, therefore, we evaluate predation pressure on butterfly communities by integrating these three complementary approaches: butterfly wing damage, artificial prey replicas, and concurrent bird surveys. We assess the reliability and effort associated with each approach and provide recommendations for their application under different ecological contexts. Here, we predicted that both wing damage and attacks on butterfly replicas would be influenced by the density and composition of the bird community as well as butterfly availability (i.e. abundance) and body size. Specifically, we predicted higher attack rates where bird diversity and density are greater, fewer attacks when butterfly abundance is high (due to a dilution effect) and more attacks on larger-bodied butterflies.

## 2. Materials and methods

### 2.1. Study sites

The study was conducted in eight sites in Sydney, NSW, Australia. Sampling was done in November, during the spring, in 2022 and 2023 (table 1).

**Table 1.** List of field sites where the study was conducted.

Year	Site name	Site code	Traditional name	Coordinates
2022	Macquarie University Campus	MQ	Wallumattagal Land	−33.77° S 151.11° E
2022	Bicentennial Park West Pymble	WPP	Cammeraygal Land	−33.63° S 151.13° E
2022	Ku-ring-gai Wildflower Garden	KRG	Kuringgai Land	−33.71° S 151.18° E
2022	Westleigh Park	WLP	Dharug and Kuringgai Land	−33.71° S 151.08° E
2023	Woo-la-ra Park	WLR	Cadigal Land and Cammeraygal Land	−33.83° S 151.07° E
2023	Parramatta Park	PP	Dharug Land	−33.81° S 150.99° E
2023	Allan Small Oval	AO	Cammeraygal Land	−33.75° S 151.18° E
2023	Jubes Mountain Bike Park	JB	Dharug Land and Kuringgai Land	−33.70° S 151.14° E

### 2.2. Butterfly sampling and identification

Butterfly nets (mesh size: 0.9 × 0.3 mm; hoop diameter: 456 mm) were used to collect butterflies with a total sampling effort of 20 h between 09.00 and 15.00 at each study site. Butterflies were collected within a short time window (within 4–5 days of each year) to reduce the variation in wing damage due to ageing, assuming similar butterfly phenology across sites. The collected butterflies were placed in glassine insect envelopes that were labelled with the date, site name, collector name, and wing damage (e.g. damage caused during collection or any damage that was already on butterfly wings). Envelopes were stored in cooler bags in the field. The envelopes with samples were taken to the laboratory and stored at −30 °C. Species-level identification was conducted for butterflies using a butterfly identification guide [33] and with the help of a lepidopterist (Assoc. Prof. Darrell Kemp, Macquarie University, NSW, Australia).

### 2.3. Scoring butterfly wing attacks

**Figure 1.**
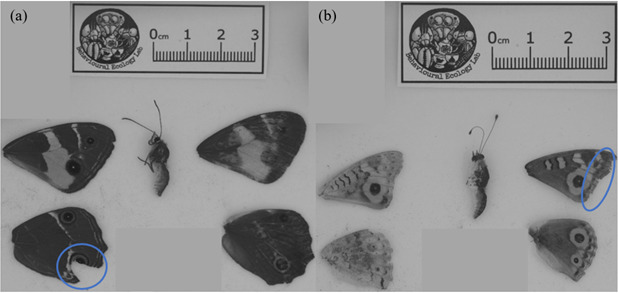
(a) Avian attack on the left hindwing of Varied Sword-grass Brown (*Tisiphone abeona*); (b) non-avian damage on the right forewing of Meadow Argus (*Junonia villida*).

Collected butterflies were photographed using a Sony A7 camera (lens: EL-Nikkor 80 mm), lit by three Exo Terra Intense Basking Spot Lamps (75 W). Wings were separated from the body and arranged against a white/black background, and a scale was included in the image (figure 1). Three people, who were unaware of the collection site, scored the damage on the wings (avian predator, non-avian predator, collection damage, or other damage) in each photograph. Avian attacks were identified based on beak imprints (U/V-shape), triangular wing tears, and straight tear cuts along the major veins on the wings [13,34–37]. To ensure consistent scoring, each scorer was trained using peer-reviewed articles [13,34,38,39]. The scores obtained from each person were collated to get the final wing attack score. In case of disagreement, the final wing attack score was based on the majority opinion (2/3) [40]. Finally, the number of butterflies attacked by birds was calculated per site.

### 2.4. Butterfly replica preparation

Staedtler Fimo® Polymer clay was used to construct the master butterfly bodies used for mould preparation (length = 1.30 cm, width = 0.30 cm). Master bodies were baked at 110 °C for 30 min. The baked master bodies were attached to a plastic container using Bostik Blu-Tack and fully covered with Barnes Pinkysil fast-set silicone. After approximately 30 min of drying time, the bodies were removed from the silicone mould cavities that were subsequently used to prepare the butterfly replica bodies. Monster Clay medium modelling clay was slowly (approx. 3 h) heated until it reached a liquid state, poured into the moulds and allowed to set at room temperature for 24 h. The cooled butterfly replica bodies were removed from the silicone moulds, trimmed using a scalpel blade, and short (2.50 cm) drawing pins were inserted into the butterfly replica bodies. Finally, the butterfly replica bodies were painted with brown acrylic paint (Mont Marte) to match the brown-coloured wings. A visible brown replica with no conspicuous marks/stripes/dots was chosen so that birds would not avoid the replica due to any resemblance to a warning signal.

**Figure 2.**
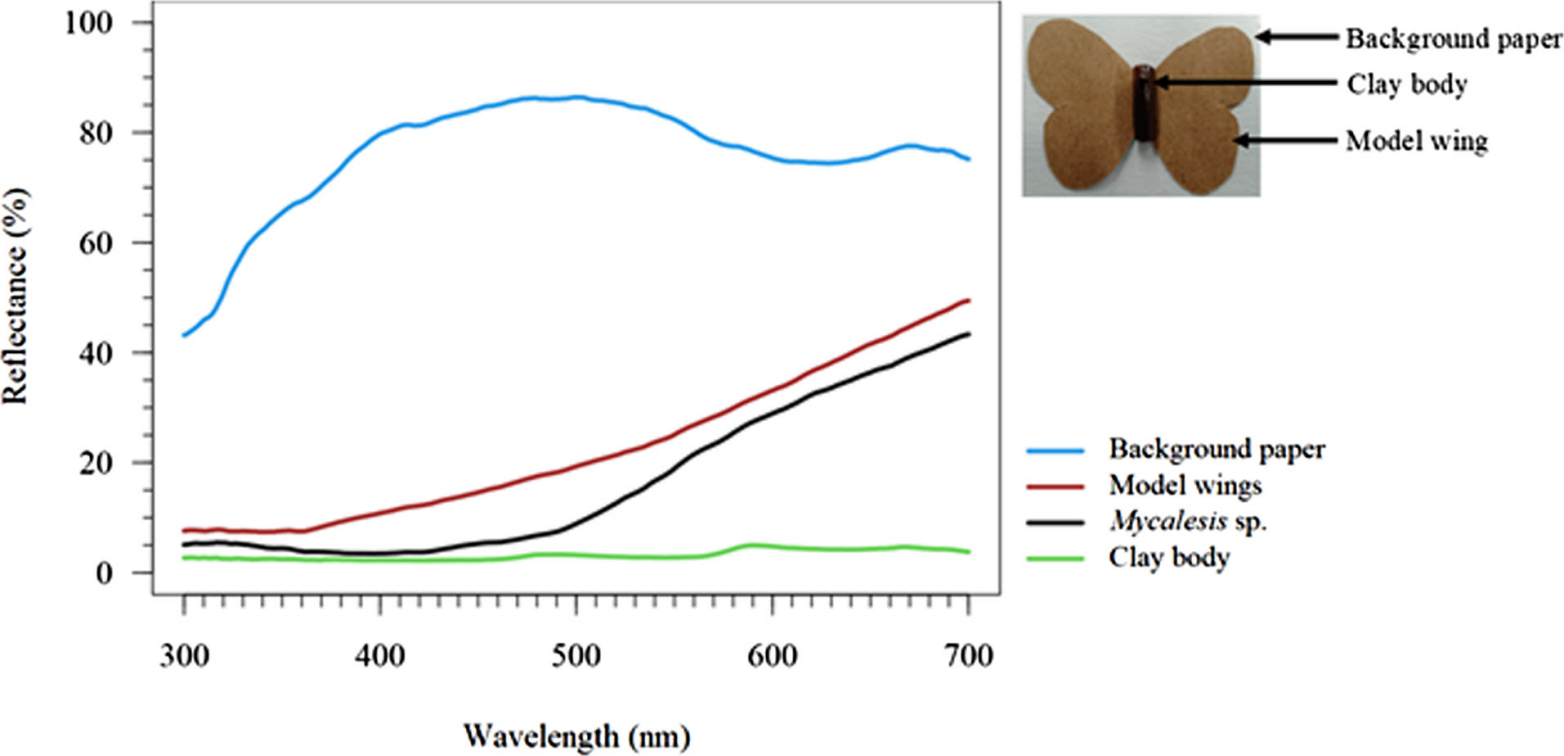
Spectral reflectance of real *Mycalesis* sp. wings, butterfly replica (model wings), replica body, and background paper. Reflectance is relative to a spectralon reflectance standard (Ocean Optics, Dunedin, Florida, USA). Colour contrast: model wing & *Mycalesis* butterfly wing: 5.9360 JND; model wing & background paper: 7.8319 JND; *Mycalesis* butterfly wing & background paper: 13.5454 JND; achromatic contrast: model wing & *Mycalesis* butterfly: 0.2772 JND; model wing & background paper: 1.0999 JND; *Mycalesis* butterfly & background paper: 1.3771 JND; colour contrast and achromatic contrast were calculated using the Blue Tit visual system (*Cyanistes caeruleus*) using PAVO [41].

The wings (4.30 cm wingspan) were made from brown paper (29.70 × 21.00 cm, Kraft Paper Pad, Anko), which we tried to match as closely as possible with *Mycalesis* sp. via spectrophotometry (figure 2) using an Ocean Optics USB-4000 spectrometer coupled with a PX-2 pulsed xenon light source (Ocean Optics, Dunedin, Florida, USA). While the achromatic contrast was below the just noticeable difference (JND) threshold (JND < 1), the chromatic contrast was above the threshold (JND > 1). We did not consider this problematic, as we only used *Mycalesis* as an approximation of a brown butterfly without conspicuous markings. The wings were then pasted on to background paper, and the clay body was glued to the wings. We selected a pale blue square background paper (4.40 × 3.30 cm) cut from Canson^®^ Mi-Teintes paper (50 × 65 cm; France) to create a standardized background with reasonable contrast to the butterfly replica (figure 2).

The wings were attached to the clay body to be open, consistent with previous experiments that commonly use this methodology (see also [29]) for two main reasons: first, to increase the detectability of the replica, and second, to allow birds to freely interact with the clay body. The downside of this design is that it does not reflect the resting position of butterflies with closed wings. We carefully considered both the advantages and disadvantages of this design and opted for the open-wing replica.

### 2.5. Butterfly replica deployment

We deployed 200 butterfly replicas along a 1 km transect at each of the eight sites, resulting in 1600 deployed replicas. Each replica was affixed to a tree trunk at a height of 1.50–2.00 m, with a maximum of one replica per tree. Butterfly replicas were positioned about 3–5 m apart, and adjacent replicas were fixed to opposite sides of the trees to prevent predators from sighting two prey replicas at the same time.

The deployment height and duration were based on the average height and duration from previous butterfly replica studies [29,42], with the aim being to minimize weather damage and fading and allow the practical deployment and retrieval of replicas.

### 2.6. Scoring butterfly replica attacks

The butterfly replicas were left exposed for 53 h, after which they were checked for attacks before being removed from the trees and returned to the laboratory. Each attacked replica was photographed using an OPPO AX7 mobile phone camera, and images were sent to three researchers with experience in clay replica damage to score the cause of the attacks (handling, birds, and other animals (mammals, reptiles, or insects)). Avian attacks were identified based on U/V-shaped beak imprints and peck marks on the clay body. Scorers were unaware of the site where the butterfly replicas were collected. Each scorer was trained using a peer-reviewed article [43]. The scores obtained from each person were collated to analyse the final replica attack score. In case of disagreement, the final replica attack score was based on the majority opinion (2/3). Finally, the number of butterfly replicas attacked by birds was calculated per site.

### 2.7. Bird surveys

Since birds are the most likely visually hunting predators of butterflies [13,37,44], bird surveys were conducted at each field site to identify birds, either by sight or call, that may feed on insects. The surveys began within two hours of sunrise and were completed within four hours of sunrise using traditional point count protocols [45,46]. Each site was surveyed twice by two bird experts, one identifier and one scribe. Five-point counts were conducted along a 1 km transect with a 50 m radius for each point per site. Points were spaced 200 m apart. The two observers spent 20 min at each point, recording all present species within the survey radius. Two steps were used to identify insectivorous birds (i.e. potential predators of butterflies). First, the bird diet was assigned using Garnett *et al*. [47]. The species that only feed on terrestrial invertebrates, according to Garnett *et al*. [47], were assumed to be true insectivorous feeders. The species that were classified by Garnett *et al*. [47] as feeding on terrestrial invertebrates and other types of food were assumed to be possible insectivorous feeders. Second, these possible insectivorous birds were further refined by a bird expert in our author team (L.G.O.) and the suggestion of an anonymous reviewer to remove species that are unlikely to feed on butterflies. Our final list of insectivorous birds included the true insectivores and the refined list of potential insectivores. The scientific names listed in Garnett *et al*. [47] were used for bird species in our study. All butterfly sampling, replica deployment, and bird surveys were conducted within 6 days on the same site.

### 2.8. Data analysis

All data analyses were performed in R (v. 4.2.2; [48]). Generalized linear mixed models (GLMMs) were used to evaluate the effects of bird and butterfly communities on avian attack rates, based on two datasets: (i) wing damage in wild butterflies and (ii) attack on artificial butterfly replicas.

**Figure 3.**
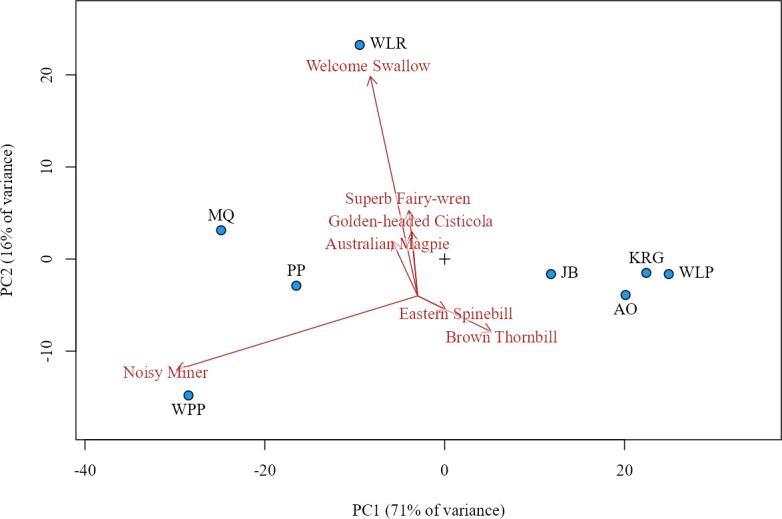
PCA of bird community composition. Points represent PC1 (*x*-axis) and PC2 (*y*-axis) scores for individual sites (electronic supplementary material, table S3). Arrows indicate bird species with the strongest loadings, showing their influence on PC axes (electronic supplementary material, table S2). Arrows are offset from the true origin (black cross) to improve visibility.

Bird density was calculated as the average number of insectivorous birds observed per site over two survey days. To summarize bird community structure, we conducted principal component analyses (PCA) using a species-by-site abundance matrix of mean insectivorous bird abundance. PC1, primarily driven by Noisy Miner (*Manorina melanocephala*), Brown Thornbill (*Acanthiza pusilla*), and Welcome Swallow (*Hirundo neoxena*), explained 70.75% of the total variation in the bird community. PC2, influenced by Welcome Swallow, Superb Fairy-wren (*Malurus cyaneus*), Noisy Miner, and Golden-headed Cisticola (*Cisticola exilis*), explained an additional 16.38%, for a total of 87.13% (figure 3; electronic supplementary material, tables S2 and S3).

Butterfly abundance was calculated for each butterfly family based on the individuals collected from each site. Butterfly species-specific and sex-specific wingspan data were obtained from *The Complete Field Guide to Butterflies of Australia* [33]. These wingspan measures were matched to each individual based on species and sex in our dataset. We then calculated the average wingspan of each butterfly family for each site.

To explore correlations among predictors, we generated a correlation matrix (pairwise Pearson correlation coefficients) and visualized it as a correlogram (electronic supplementary material, figure S1). Variables included PC1, PC2, density of the insectivorous birds, butterfly abundance and average wingspan, and the six most abundant insectivorous bird and butterfly species.

### 2.9. Wing attack analysis

Our data were aggregated by site and butterfly family, resulting in 32 observations. We fitted a series of GLMMs to examine predictors of avian attack on butterfly wings. The response variable was the binomial count of attacked versus non-attacked butterflies per site. Fixed effects included: bird community composition (PC1 and PC2), bird density, butterfly abundance, and butterfly wingspan. Site and butterfly family were included as random effects in the initial model.

GLMM 1 (electronic supplementary material, table S4; Akaike information criterion (AIC): 131.22) included all fixed and random effects. However, both random effects had zero variance, indicating they did not improve model fit. Single-term deletion analysis (electronic supplementary material, table S5) showed that excluding butterfly abundance improved model performance (AIC reduced 129.23 versus 131.22), leading to GLMM2 (electronic supplementary material, table S6). In GLMM 2, fixed effects were PC1, PC2, bird density, and butterfly wingspan, with site and butterfly family as random effects (both still with zero variance). This model was further simplified by removing the random effect butterfly family, resulting in GLMM 3 (electronic supplementary material, table S8), which retained only the site as a random effect (again, with variance = 0). To address singular fit issues and improve model stability, GLMM 4 (table 2) log-transformed bird density and butterfly wingspan. This transformation retained explanatory power and maintained the lowest AIC (127.16), suggesting it was the best-fitting model overall. Random effects again showed zero variance, indicating minimal site-level structure in predation rates.

**Table 2.** GLMM 4 showing the effect of fixed effects: PC1, PC2, and density of insectivorous birds, and butterfly wingspan (AIC: 127.16). The variation and standard deviation of the random effect (site) are 0.0163 and 0.1276.

Source	Estimate	S.E.	Z	*p*
Intercept	−1.6308	0.1155	−14.1210	< 0.001 ***
Bird community (PC1)	0.4542	0.1329	3.4180	< 0.001 ***
Bird community (PC2)	−0.5986	0.1520	−3.9390	< 0.001 ***
Bird density (log)	0.3752	0.1214	3.0910	0.002 **
Butterfly wingspan (log)	0.5900	0.1461	4.0390	< 0.001 ***

### 2.10. Butterfly replica attack analysis

Our data were aggregated by site, resulting in eight observations. We fitted a series of GLMMs to assess predictors of attacks on artificial butterfly replicas. The response variable was the binomial count of attacked versus non-attacked replicas per site. The full model (GLMM 5, electronic supplementary material, table S9; AIC: 39.473) included four fixed effects: bird community composition (PC1 and PC2), insectivorous bird density, and butterfly abundance (accounts for the potential variation of natural prey abundance at the time of replica deployment). The site was included as a random effect, and the variance was zero. Single-term deletion analysis (electronic supplementary material, table S10) indicated that removing butterfly abundance improved model fit (AIC: 37.881), leading to GLMM 6 (electronic supplementary material, table S11). In this model, the site had zero variance. Further deletion of bird density and retaining only PC1 and PC2 as fixed effects produced the final model with the lowest AIC and best fit (GLMM 7; table 3; AIC: 37.061; the site retained zero variance) [49].

**Table 3.** GLMM 7 showing the effect of fixed effects: PC1 and PC2 of insectivorous birds (AIC: 37.061) on butterfly replica attacks. Variation and standard deviation of the random factor site: 0.

Source	Estimate	S.E.	*Z*	*p*
Intercept	−4.0883	0.2171	−18.828	< 0.001 ***
Bird community (PC1)	0.6020	0.2479	2.428	0.0152 *
Bird community (PC2)	0.3215	0.2153	1.493	0.1353

## 3. Results

### 3.1. Butterfly community composition

We collected a total of 1070 butterflies across eight field sites in Sydney, with 329 collected in 2022 and 741 in 2023 (see electronic supplementary material, table S14 for the list of species). Most individuals were males (69%), and specimens represented 38 species across five families: Pieridae, Lycaenidae, Nymphalidae, Hesperiidae, and Papilionidae. Pieridae accounted for the largest proportion of the sample (39.35%), followed by Lycaenidae (34.21%), Nymphalidae (17.48%), Hesperiidae (8.79%), and Papilionidae (0.19%). The highest butterfly species richness was recorded in Jubes Mountain Bike Park, while the highest butterfly abundance was recorded in Parramatta Park (table 4).

**Table 4.** Butterfly richness and abundance, bird richness and density, wing attacks, and butterfly replica attacks in eight sampling sites in Sydney, Australia: MQ—Macquarie University Campus; WPP—Bicentennial Park West Pymble; KRG—Ku-ring-gai Wildflower Garden; WLP—Westleigh Park; AO—Allan Small Oval; JB—Jubes Mountain Bike Park; WLR—Woo-la-ra Park; PP—Parramatta Park.

**Site code**	**Butterfly species richness**	Butterfly abundance	Bird species richness	Bird density (individuals km^−2^)	Wing attacks (%) total *n* = **169**	Butterfly replica attacks (%) total *n* = **31**
MQ	11	55	14	2686.53	23.64	1.50
WPP	12	46	07	1858.93	15.22	0.50
WLR	10	187	15	2737.47	4.81	2.50
PP	10	290	11	1909.86	11.72	0.50
KRG	13	158	17	1285.97	20.25	4.00
WLP	13	70	22	2113.58	22.86	2.00
AO	05	144	21	2049.92	20.83	3.50
JB	16	120	24	2571.94	23.33	1.00

### 3.2. Bird community composition

We recorded 83 species of birds during our survey period. For analyses, we included only 49 species that are at least partially insectivorous (see bird survey methods and electronic supplementary material, table S2). The highest bird species richness was recorded in Jubes Mountain Bike Park, and the highest bird density was recorded in the Woo-la-ra Park (table 4).

### 3.3. Bird predation on actual butterflies and butterfly replicas

Of the 1070 butterflies collected, 807 individuals (75% of all butterflies collected) exhibited some form of damage to their wings. Damage was categorized as Avian: marks characteristic of bird attacks; Non-avian: predation by other types of predators, such as lizards, mammals, or invertebrates; Collection: damage caused by butterfly net or handling of the butterfly; Other damage: wear and tear possibly from flight, ageing or unknown mechanical causes. Among those, 169 individuals (16% of all butterflies collected) were assessed as having wing damage caused by birds. Three scorers agreed on 55% of avian attack cases, and two scorers agreed on the remaining 45% of avian attack cases. While we captured many more males (68.41%) than females (31.50%), attack rates were very similar (male: 15.71%; female: 16.02%).

Among 1600 butterfly replicas, 114 (7%) were attacked or damaged. Of those, we considered 31 butterfly replicas (2% of all models) to have been attacked by birds. Three scorers agreed on 32% of avian attack cases, and two scorers agreed on the remaining 68% of avian attack cases.

### 3.4. Avian wing attacks on wild butterflies

Our logistic regression model for butterfly wing attacks aimed to explain how the insectivorous bird community and butterfly community affected the likelihood of butterfly wing attacks by avian predators (GLMM 4; table 2). Wing attacks were significantly and positively explained by insectivorous bird PC1 and bird density, while bird PC2 showed a significantly negative relationship to the wing attacks. Butterfly wingspan showed a significant positive correlation to wing attacks. Butterfly abundance and butterfly family did not show significant relationships with wing attacks (GLMM 1; electronic supplementary material, table S2).

### 3.5. Butterfly replica attacks

The logistic regression model for artificial butterfly replica attacks explained how the insectivorous avian predator community and real prey (butterfly) community affected the likelihood of butterfly replica attacks caused by avian predators (table 3). Both PC1 and PC2 of birds were significantly and positively related to the butterfly replica attacks. Bird density, butterfly abundance, and butterfly family did not show any significant relationships to the butterfly replica attacks (GLMM 5: electronic supplementary material, table S9; GLMM 6; electronic supplementary material, table S11).

## 4. Discussion

This study compared multiple complementary methods for estimating avian predation pressure on butterfly communities. Rather than focusing on a single approach, we assessed how well wing damage rates in wild butterflies, attack rates on artificial replicas, and bird and butterfly community data align and complement one another. By cross-validating these approaches, we aimed to identify which metrics offer the most reliable insights into real-world predation and under what ecological contexts they are most informative. To the best of our knowledge, this is the first empirical study to simultaneously evaluate these different methods in butterflies, providing methodological insights and practical recommendations for future studies.

The composition of the predator community has been shown to significantly influence the likelihood of bird attacks on caterpillars, moths, and even snakes [50–53]. Consistent with our hypotheses, we found that variation in bird communities significantly predicted both wing damage rates and replica attack rates. We also predicted that higher bird density would increase predation pressure, and this was supported for wing attacks but not for replica attacks. In contrast to our expectations, butterfly abundance did not predict attack rates. However, as predicted, larger butterflies were more likely to be attacked (see also [24,54]).

Our results underscore the importance of not just bird abundance, but bird community composition and functional traits for understanding variation in predation risk and attack patterns on butterflies. The significant relationship between wing attacks and bird PC1 and PC2, and bird density in our study implies that the presence of certain bird–predator assemblages and higher bird–predator abundance are more likely to increase the risk of predation on butterflies. This shows that some bird species are effective predators of butterflies, while others might have different, less effective feeding behaviours, foraging on other prey or their presence may exclude other insectivorous bird predators. For instance, Noisy Miners, a dominant species in PC1, are highly territorial and actively exclude other birds from their territory [55–57], potentially lowering predation pressure at sites where they are common. Alternatively, Noisy Miners may pursue and eat whole butterflies more persistently than other bird species, which may have resulted in an underestimation of predation events in sites with Noisy Miners. Their flexible foraging behaviour, including a shift toward nectar and human-provided food [58], may also explain the low predation signals associated with them.

In contrast, Brown Thornbills, also loaded significantly on PC1, are primarily insectivorous and less territorially aggressive [59,60], possibly allowing for higher wing attacks. Welcome Swallows, which are aerial insectivores, were associated with fewer attacks, probably due to limited interaction with butterflies. Conversely, species like the Grey Butcherbirds are active insectivorous predators and may drive higher wing attack rates. Our data suggest that not all insectivorous bird species exert equal pressure on butterflies. Birds vary greatly in foraging style, spatial niche and prey handling strategies. By incorporating predator functional guilds into analyses, future work could refine estimates of predation pressure and better capture how specific bird foraging strategies align with butterfly behaviours and microhabitats.

Beyond the methodological validation, our results offer biologically meaningful insights into the ecological and evolutionary dynamics of butterfly–bird interactions. We predicted that both prey availability (abundance) and size would influence wing attacks. We did not find evidence for the significant effect of butterfly abundance on wing attacks, but we did find an effect of wingspan, with larger butterflies at greater risk of bird predation. This is similar to other studies that have shown that larger butterflies are more likely to receive bird attacks [24,54].

This increased vulnerability with larger body size can be due to various factors. This reflects that larger-bodied butterflies are more easily detectable by avian predators and provide more nutritional value aligned with optimal foraging theory [32,61]. Heavy abdomens, differences in body mass centre and wing morphology, slower flight speeds, and poor manoeuvrability also cause a lack of escape ability from predators [21,32,62]. In contrast, this result could also be due to survivorship bias of larger butterflies after receiving bird attacks. Over evolutionary timescales, such selection pressures may drive shifts in body size distributions within populations or favour the development of compensatory traits such as disruptive patterning, faster escape responses or mimicry. These findings highlight the role of predator community structure as a selective force shaping morphological traits in butterfly communities.

To the best of our knowledge, our study is the first to relate artificial butterfly replica attacks to bird predators and actual prey communities. Similar to our wing damage data, bird community composition (but not density) significantly predicted replica attacks. As for wing damage, the presence of Noisy Miners was associated with fewer replica attacks, indicating they were not engaging with replicas and/or their territorial behaviour excluded potential bird predators of replicas. Interestingly, replicas and wing attacks were not significantly correlated (electronic supplementary material, table S15), suggesting these methods capture different aspects of predation. This probably reflects the limitations of replicas: they lack chemical cues, movement and behavioural context, and may elicit exploratory pecks rather than genuine feeding behaviour. Still, replicas offer a controlled, standardized method useful for broad-scale comparisons.

### 4.1. Methodological considerations

Both methods (replicas and wing attacks) have advantages and limitations. The wing damage analysis is less labour-intensive and simultaneously provides butterfly community data (diversity and density) than preparing and deploying replicas. However, using wing damage could miss some predation attacks if the collection does not cover the entire activity period of butterflies in the community, and it cannot identify butterflies that were entirely eaten. Another key limitation of the wing attack method is the potential subjectivity of wing damage scoring. While previous studies [13,34–37] have identified typical beak-shaped patterns on butterfly wings from bird attacks, predation can also be from lizards or mantids, which may cause similar wing damage. Moreover, minimal damage from failed attacks may be missed or misclassified. In our study, we applied conservative classification criteria and excluded uncertain cases to minimize misinterpretation. Undoubtedly, this will have created some biases, but these would be consistent across sites and would still allow a meaningful comparison between sites.

Replica attacks, while standardized and scalable, lack behavioural realism. Their static posture and artificiality may reduce ecological validity (see also [29,42]). Despite this, they remain a valuable tool, especially when natural predation is difficult to observe and could be improved with the addition of camera traps to verify attackers.

Bird surveys are low in effort and provide useful information about dominant species, such as Noisy Miners, that can then be considered in the interpretation of the data. Bird surveys require experts and only provide a temporal snapshot, although this limitation can be mitigated by resampling over several days. Bird diversity data need to be interpreted cautiously, as birds classified as ‘insectivorous’ may feed on a range of invertebrates [63] and may thus not represent an actual predator threat to butterflies, and/or they may switch food sources at different times of the season [64–66]. Nevertheless, we consider that bird surveys were informative in conjunction with actual wing attack data and replica attacks.

Finally, since bird and butterfly diversity and abundance vary with season, it is important to consider a suitable time of the year for a survey and ensure all sites are sampled at a similar time. We sampled during the Australian spring both years, when butterfly and insectivorous bird activity was at its peak. We also followed the same time window for our sampling in both years and sampled all sites within one week. Thus, we believe that variation in timing has a minimal impact when comparing between sites.

## 5. Conclusion

Our results strongly suggest that wing damage and replica attacks reflect different characteristics of predation pressure by birds. Wing damage represents real but failed attacks under natural conditions and incorporates behavioural, sensory and ecological cues, though it may miss successful predation events. Replica attacks, in contrast, reflect potential visual risk but lack biological realism. We argue that integrating multiple methods, especially paired with bird community data, provides a more robust estimate of predation pressure in natural systems. Moreover, understanding which bird species are present and how they behave is essential for interpreting these data meaningfully. We advocate for further comparative studies across predator–prey systems to test the generality of these findings and to refine our tools for measuring predation in the wild. Our results underscore the ecological and evolutionary importance of avian predation in structuring butterfly communities and call for more biologically integrated approaches in future research.

## Data Availability

The data underlying the work are available in the main article and supplementary materials [67]. Additionally, some of the butterfly images collected during the study are accessible via Zenodo at: https://doi.org/10.5281/zenodo.15881961.

## Authors' Notes

We respect and support diversity, equity, and inclusion in science. The authors originated from different countries (Australia, Austria, Brazil, Finland, France, Sri Lanka, United Kingdom, and United States of America), representing different backgrounds, cultures, ethnicities, genders, and career stages (Ph.D. candidates, postdoctoral fellows, early and mid-career researchers, and professors). Our author sex ratio is female-biased, and at least one author identifies as a sexual minority or a person with a disability.
